# Perceived job insecurity and risk of suicide and suicide attempts: a study of men and women in the Swedish working population

**DOI:** 10.5271/sjweh.4015

**Published:** 2022-04-29

**Authors:** Sandra Blomqvist, Marianna Virtanen, Anthony D LaMontagne, Linda L Magnusson Hanson

**Affiliations:** 1 Stress Research Institute at Department of Psychology, Stockholm University, Stockholm, Sweden; 2 School of Educational Sciences and Psychology, University of Eastern Finland, Joensuu, Finland; 3 Institute for Health Transformation, and School of Health & Social Development, Deakin University, Geelong, Victoria, Australia. ORCID 0000-0002-5811-5906

**Keywords:** epidemiology, job stress, labor market condition, longitudinal study, mental health, mortality

## Abstract

**Objective:**

Whether perceived job insecurity increases the risk of suicidal behaviors is unclear. Improved understanding in this area could inform efforts to reduce suicide risk among those experiencing elevated job insecurity during the COVID-19 pandemic as well as post-pandemic. We aimed to investigate if perceived job insecurity predicted increased risk of suicide mortality and suicide attempts.

**Method:**

Employees (N=65 571), representative of the Swedish working population who participated in the Swedish Work Environment Survey in 1991–2003, were followed up through 2016 in the National Inpatient and Death Registers. Suicide deaths and suicide attempts were defined according to International Classification of Diseases (ICD) 10 and ICD-8/9 codes of underlying cause of death and in-/outpatient care. Job insecurity and subsequent risk of suicide and suicide attempt were investigated with marginal structural Cox regression analyses and inverse probability of treatment weighting to control for confounding.

**Results:**

Perceived job insecurity was associated with an elevated risk of suicide [hazard ratio (HR) 1.51, 95% confidence interval (CI) 1.03–2.20], but not with incident suicide attempts (HR 1.03, CI 0.86–1.24). Estimates remained similar after considering prevalent/previous poor mental health, other work factors, and when restricting the follow up time to ten years.

**Conclusion:**

The study suggests that job insecurity is associated with an increased risk of suicide mortality. Concerns about elevated job insecurity and suicide levels in the wake of the current pandemic could thus be considered in strategies to reduce the population health impact job insecurity both during and following the COVID-19 pandemic.

Suicide is a serious and tragic event with long-term effects for family and friends as well as the communities left behind. Although age-standardized global suicide rates have remained fairly stable or even declined slightly over the last two decades (1, 2), an increasing trend in suicide deaths has been seen among adolescents and young adults, which has been a cause for concern in Sweden and worldwide (3–5). It is a public health priority to turn this upward trend around.

Research on work-related psychosocial factors and suicidal behaviors is limited, especially large-scale prospective cohort studies (6). A greater understanding of how working conditions affect suicidal behavior is of importance as a large proportion of all suicide attempts and deaths occur among working age individuals (1).

Job insecurity is defined as a subjective perception of the threat of or concern for job loss, it is a widely prevalent psychosocial work stressors associated with stress/distress (7). Job insecurity is likely increasing due to labor market impacts of the current COVID-19 pandemic, potentially adding to or compounding other pandemic-related mental health impacts (8). Generally, work stressors, including job insecurity, are assumed to contribute to an increased risk of mental health problems, poor physical health or both, by eliciting elevated levels of stress causing a wear and tear on the bodily stress regulating functions and/or by making individuals more prone to adopt risky health behaviors (9). Other potential pathways through which job insecurity could lead to mental health problems (10) include lowered self-worth (11), violation against expectations regarding one’s employment continuance (ie, psychological contract breach) (12), and/or perceptions of not being in control over one’s work situation (13).

Previous studies have found support for an association between job insecurity and an elevated risk of psychological morbidity (7, 14), in particular depression (15), and of physical morbidity, including diabetes, and heart disease (7, 14). In addition, associations between job insecurity and metabolic markers including urinary catecholamines, cholesterol, blood pressure and body mass index (BMI) have been found in earlier studies (7). Job insecurity has also been suggested to be one of the strongest work-related risk factors for suicide ideation (16). However, far from all ideators progress to action (17) and predictors of ideation may differ from predictors of suicidal behaviors such as suicide attempt and suicide (both associated with the capacity to take action).

Stressful events have generally been proposed as potential precipitating/proximal factors for suicidal behavior (18) and poor social support and low control at work may be associated with suicide attempt or suicide death, according to a systematic review of the psychosocial job stressor research literature (16). Suicide rates have been shown to rise in the wake of economic crises associated with a higher level of layoffs and redundancies (19), which may be partly attributed to job insecurity (20). Perceived job insecurity has been associated with national and regional unemployment rates (21, 22), and unemployment increases the risk of suicide both on an aggregated (19) and individual level (21). There is a concern for increased suicide rates in the wake of the COVID-19 pandemic partly due to loss of employment and associated financial loss (23), and there has been a call for research that can contribute to strategies reducing the potential mental health consequences of the pandemic (24). However, few studies have investigated whether perceived job insecurity increases the risk of suicide attempts and suicide deaths directly and, findings have been inconclusive (16).

To strengthen the evidence on this topic, we performed a prospective cohort study on perceived job insecurity and risk of suicide and suicide attempt in a large sample of Swedish men and women.

## Methods

### Study sample

The present study is based on participants in the Swedish Work Environment Survey (SWES) 1991–2003, a nationally representative biennial cross-sectional survey based on a subsample of the Labor Force Survey (LFS). After simple random sampling and stratification for county, sex and age, a subset of respondents to the LFS, aged 16–64 and in paid work were asked to respond to self-completion SWES questionnaires. In 1991, it was estimated that about 90% of people in work responded to the LFS. In SWES, about 64–82% of all invited participants responded, with decreasing response rates over time, resulting in a sample of 74 082 unique individuals with information on job insecurity at one occasion taken some time during the years 1991–2003. After excluding people with invalid identification numbers and those with inappropriate data on age at end of follow-up, or invalid data on covariates, the total study sample included 65 571 respondents.

The Regional Research Ethics Board in Stockholm approved the study. Participants of the SWES received written information on the survey, and informed consent was confirmed by answering and returning the survey.

### Study variables

Job insecurity was assessed with the following SWES question: *Are you under threat of temporary or permanent dismissal?* Respondents were considered exposed to job insecurity if reporting to be under threat of dismissal, otherwise unexposed. Suicide attempts and deaths were identified from the Swedish Patient Register, including both in- and outpatient data (since 2001) and the Causes of Death Register. Suicides were ascertained by a diagnosis of X60-X84 (self-inflicted harm) or Y10-Y34 (death with undetermined intent) in the International Classification of Diseases (ICD) 10 version or E950-959 and E980-989 in ICD-8/9 as underlying cause of death. Suicide attempts were ascertained by a diagnosis of the corresponding codes of self-inflicted harm or injuries with undetermined intent in the Patient Register. Although we did not have any prior hypothesis that the causal pathways from job insecurity to suicide attempt or death would differ, we used suicide attempts and deaths as distinct outcomes, as advised in order to enable progress in theoretical models and knowledge about suicidal behaviors (17).,

Information about sex, age, family situation, country of birth, income and educational level (see table 1) were collected from the longitudinal integration database for health insurance and labor market studies (LISA) and treated as potential confounders. Apart from income, the above-mentioned covariates were associated with both job insecurity and suicide deaths/attempts and therefore included as confounders in the statistical models. In an attempt to rule out reverse causation from poor mental health, ie, that poor mental health affected the reporting or experience of job insecurity and also increased the risk for suicidal behavior, we additionally considered poor mental health up to baseline assessed by baseline reports of being tired or listless every day or having a history of prior mental disorders defined by diagnoses of ICD-10 F01-99 or ICD-9/ICD-8 290-319. Similarly, we considered other work-related stressors collected in the SWES that might be related to both the exposure and the outcome. These included job demands and control, measured by indices for demands and control based on four items each scored 0–4, along with an index for support at work measured with two separate questions regarding support from superiors and fellow workers scored 1–4. Measures of poor mental health and work-related stressors were considered in sensitivity analyses as they are potential confounders but could also be mediators of the relationship between job insecurity and suicidal behavior. Finally, we considered certain physical disorders such as asthma, cancer, chronic obstructive pulmonary disease, coronary heart disease, diabetes, and stroke, ascertained from in- and outpatient data. These were, however, not associated with job insecurity and therefore not included as confounders.

**Table 1:** Distribution of sociodemographic factors according to job insecurity. [SD=standard deviation; SEK=Swedish krona).

	All			Not exposed to job insecurity			Exposed to job insecurity		
		
	N	%	Mean (SD)	N	%	Mean (SD)	N	%	Mean (SD)
Sex									
Men	30 967	47		26 862	47		4105	48	
Women	34 604	53		30 080	53		4524	52	
Age			42 (12)			42 (12)			40 (11)
Birth country									
Nordic countries	63 236	96		54 970	97		8266	96	
Elsewhere	2335	4		1972	3		363	4	
Family situation									
Married/living with partner w/ children	31 351	48		27 187	48		4164	48	
Married/living with partner w/o children	11 353	17		10 151	18		1202	14	
Single/divorced/separated/widowed w/ children	4975	8		4261	7		714	8	
Single/divorced/separated/widowed w/o children	17 892	27		15 343	27		2549	30	
Education									
Compulsory school (≤9 year)	13 203	20		11 511	20		1692	20	
Gymnasium (10–12 year)	32 727	50		27 890	49		4837	56	
University (≥13 year)	19 641	30		17 541	31		2100	24	
Yearly income from work in SEK			196 528 (111 252)			199 779 (111 788)			175 070 (105 161)
Job demands	1.7	1.3		1.7	1.3		1.6	1.3	
Job control	2.5	1.3		2.5	1.3		2.3	1.3	
Support at work	1.6	1.0		1.6	0.9		1.7	1.0	

### Data analyses

We followed respondents from baseline, ie, the year of responding to the SWES questionnaire, until incident suicide attempt, suicide, or until the respondent migrated, died by another cause, or reached the end of follow up (2016).

Separate analyses for the risk of suicide death and suicide attempts were performed using marginal structural Cox (MSC) models with inverse probability of treatment weighting (IPTW), providing hazard ratio (HR) estimates. In the analyses of suicide attempt, respondents with previous suicide attempts were excluded. Stabilized IP weights were calculated in two steps (25). First, a logistic regression model conditioned on age, sex, family status, country of birth and education, were fitted to calculate each individual’s inversed probability of being exposed to job insecurity, generating a re-weighted sample, a pseudo-cohort, with better balance over covariate strata between exposed and unexposed. Secondly, weighted Cox proportional hazard models were fitted using the weights from the previous step with age as the underlying time scale. Given that the included covariates were correctly identified and specified, MSC models with IPTW provide marginal HR, which is comparable to contrasting risks of suicide death or attempt in a pseudo-population where everyone is exposed to a pseudo-population where no one is exposed. This contrast improves the ability to make causal interpretations according to the potential outcome approach. We used bootstrapping with a resampling of 500 to derive more accurate standard errors and confidence intervals (CI) with 95% coverage. In sensitivity analyses, we further added baseline poor mental health, and work characteristics in the models. The analyses were also stratified by sex, and an interaction term was included to assess if the associations differed between men and women, by age and educational level. Furthermore, sensitivity analyses of suicide attempts and death excluding diagnoses with undetermined intent were performed to assess the robustness of the findings. Lastly, models with a maximum follow-up time of ten years and with traditional Cox regression analysis were performed for comparison. No violation of the proportional hazard assumption was found after tests of interaction terms between time and exposure and a visual inspection of log-log. The analyses were performed with SAS 9.4 (SAS Institute, Cary, NC, USA).

### Role of the funding source

The funder of this study had no role in study design, data collection, data analysis, data interpretation, or writing of the report. The corresponding author and the last author had full access to all data in the study. All authors were independent from the funder, decisions were taken unrestrictedly by the authors, who further had final responsibility for the decision to submit for publication.

## Results

Table 1 presents descriptive statistics. In total, 8629 (13%) reported job insecurity, with similar proportions for men and women. Distribution of sociodemographic factors and work conditions were fairly similar across the exposed and unexposed, although the exposed had somewhat lower education level, income and job control. In particular, lower education and job control was seen among women with job insecurity, while lower income and job control was seen among men with job insecurity (supplementary material, www.sjweh.fi/article/4015, table S1). A somewhat higher proportion of exposed were also single, especially single without children among men and single with children among women, a pattern that was particularly pronounced for exposed suicide cases (data not shown).

The participants were followed for a total of 1 223 539 person years (mean 19 years). In total, 170 persons (0.3%) died by suicide during follow-up (rate 0.1 cases per 1000 person years), 33 (0.4%) among exposed and 137 (0.2%) among unexposed to job insecurity. The MSC model with IPTW adjusting for potential confounding by sex, birth country, family situation, and educational level, showed a HR of 1.49 (95% CI 1.01–2.20, table 2). Estimates from the traditional Cox model for suicide, adjusted by the same factors, gave a HR of 1.51 (95% CI 1.03–2.20, supplementary table S2). The MSC models additionally considering previous poor mental health and work characteristics gave similar albeit slightly weaker estimates (HR 1.46, 95% CI 0.99–2.16 and HR 1.44, 95% CI 0.97–2.14, respectively). No interactions by sex, age or educational level were noted. However, the sex-specific estimate was somewhat higher for women (HR 1.90, 95% CI 0.97–3.73) than men (HR 1.29, 95% CI 0.77–2.17). MSC analyses based on a follow-up limited to ten years supported an association (table S2, HR 1.93, 95% CI 1.14–3.27, with relatively similar estimates for men and women). A HR of 1.96 (95% CI 1.14–3.38) was observed when additionally adjusting for work characteristics and previous poor mental health (data not shown). The MSC estimates were similar but mostly more conservative than corresponding estimates from traditional Cox regression analyses considering the same covariates (tables S2 and S4).

**Table 2:** Association between job insecurity and suicide attempts and suicide mortality.^a^ [HR=hazard ratio; CI=confidence interval.] **Bold indicates significance at an alpha level of 0.05**.

	N	Cases	HR	95% CI
Suicide				
All	65 571	170	**1.49**	**1.01–2.20**
Men	30 967	114	1.29	0.77–2.17
Women	34 604	56	1.90	0.97–3.73
Suicide attempts			
All	65 330	896	1.03	0.86–1.24
Men	30 857	439	1.20	0.93–1.54
Women	34 473	457	0.88	0.68–1.14

a Estimates obtained from marginal structural Cox regression analyses with inverse probability weighting considering sex, birth country, family situation, and educational level.

In the subsample excluding individuals with previous suicide attempts (N=65 330, 1 211 613 person years remaining in analysis), 896 (1%) persons had an incident suicide attempt (rate 0.7 cases per 1000 person years); 129 individuals (1.5%) among exposed and 767 (1.4%) among unexposed. No association between job insecurity and suicide attempts was observed in the main analyses (table 2, HR 1.03, 95% CI 0.86–1.24), nor in the sensitivity analyses excluding deaths of undetermined intent (supplementary table S5), or with a shorter follow-up time (HR 0.99, 95% CI 0.76–1.29, table S3). Furthermore, there were no major differences in sensitivity analyses considering previous poor mental health and other work characteristics and no indication of interaction with sex, age or educational level.

## Discussion

The findings of this prospective cohort study suggested a relationship between job insecurity and suicide but not with incident suicide attempt. The association magnitude increased after limiting the follow-up time and thus the risk of having experienced other major life events which could have affected the exposure and outcome association.

This study adds to a relatively recent review study on psychosocial job stressors and suicidality (16), which showed that job insecurity was associated with higher odds of suicide ideation. However, none of the included studies had examined job insecurity in relation to suicide attempts and only one study on job insecurity and death by suicide was identified (26). This study did not find an increased risk for suicide but was based on a total sample of only 175 individuals (26). Our findings are in accordance with a qualitative study which focused on male construction workers and observed that many of the suicide decedents had previous circumstances of job insecurity and transient job experiences (27). Previous studies have found an association between unemployment and suicide, particularly among men (28). An excess risk of suicide death in relation to job loss following workplace closure and mass lay-offs, likely associated with preceding fear of job loss, have been found, among men in particular (29) but also for men and women alike (30).

Our analyses yielded no support for an association between job insecurity and suicide attempts which was unexpected in the light of estimates for suicide and previous findings on suicidal ideation (16). To our knowledge there are no previous published studies investigating perceived job insecurity and suicide attempt. However, a relationship with suicide but not suicide attempts has been observed in some studies looking at stressors such as negative life events (31). It has also been found that job stress and financial problems are more common among people who die by suicide compared to suicide attempters (32). Our findings, together with the studies mentioned above, would thus conform to previous suggestions that individuals who make non-fatal suicide attempts and individuals who die by suicide share many characteristics but also represent distinctly different groups (33–35). Therefore, a distinction in suicidal behaviors is not necessarily limited to that of ideators and individuals with suicidal behaviors, it is seemingly fruitful to distinguish between different types of behaviors (17). To date it is not completely clear though what these distinct characteristics are. One difference that is often brought forward is the presence of prior suicide attempts. Despite being a strong predictor of both later attempts and death by suicide, suicide attempters generally seem to have a more frequent history of non-fatal attempts, which has been interpreted as reflecting a more chronic type of psychological morbidity among those who attempt suicide versus those who carry it out (32, 34). This association was, however, not replicated in another study comparing suicide attempts and deaths (33). Another difference, previously brought forward, concerns differences in health behaviors. Compared to suicide attempters, people who die by suicide more often seem to have been diagnosed with alcohol dependence (33) and having consumed alcohol or substances in connection with their suicide, potentially influencing determination as well as judgement (32). However, previous meta-analyses have shown that substance use disorders and prior alcohol use were associated with suicide ideation, attempts and deaths (36, 37). High alcohol intake among individuals reporting exposure to job insecurity has been found to be a reflection of pre-existing drinking behaviors (38). However, in general, relatively little is known about job insecurity and health behaviors (7). This points to the need for more studies investigating the relationship between job insecurity and suicidal behaviors with large enough samples in order to distinguish between suicidal thoughts, attempts and deaths as well as self-harm as distinct from suicide attempts. Such research should, preferably, also account for exposure to early life events occurring in sensitive periods or an accumulation of exposures associated with an increased risk of ill health and harmful behaviors.

Job insecurity is usually considered an aspect of the psychosocial work environment that can be associated with psychobiological changes and risk of disease/disorders (9), and in most theoretical models that aim to explain suicidal behavior, the presence of stress is central (39). Several models suggest a likely interplay between distal and proximal factors. Certain distal factors – such as personality traits, genetics, or early life adversities that create a specific diathesis or vulnerability – may predispose some individuals to suicidality, while factors such as financial and occupational difficulties, together with other negative life events and stress outside the work sphere, may increase the risk of suicide by acting as triggers (31, 32). Distal and proximal factors in combination may thus explain why some people attempt or die by suicide. Job insecurity has previously been linked to depressive symptoms (7) and mental disorders are important risk factors for suicide and serious suicide attempts (3).

Previous studies have shown that the risk of suicide attempt is higher among women while the risk of suicide death appears to be higher among men (3, 17, 32, 35). Higher risk of suicide death has further been found among men in connection with a relaxation in employment protection legislation (40) and job loss (29) in some studies. Typically, the male breadwinner ideology (primary provider) has been used as a potential explanation (41). The applicability of this explanation in more equal societies has been questioned, however, as a reduced breadwinner culture may contribute to more similar associations for men and women (41). For instance, similar effect estimates were found among young women in Sweden compared to men concerning their risk of suicide after job loss (42). In more recent studies, a stronger relationship has been found between female unemployment and suicide (43) but also a stronger association between job insecurity and poor mental health for men than for women (44). In the present study, we did not find any statistically significant differences between men and women. This, together with the somewhat inconclusive findings in the studies mentioned above concerning the role of gender on the risk of suicide following job loss and the role of gender as a potential moderator remain unclear within the broader job insecurity literature (7). Further studies aiming to understand potential gender differences in associations of job insecurity and suicide are needed.

### Strengths and limitations

The present study used Swedish health registers with good coverage and high validity with regard to ascertainment of suicide deaths and attempts (45). Outcome detection via register further precludes recall and social desirability bias. Moreover, the study was basically free from loss to follow-up and followed a representative population-based sample not limited to a specific company or industry over a long time. The long follow-up may have contributed to increased power but on the other hand a dilution of the effects of exposure. There is evidence of both short-term and cumulative effects of job security on mental health (44), but our study considered several different follow-up times. However, as the follow up time is limited so are the number of cases of suicide attempts and deaths. Therefore, our analysis with ten years of follow-up may have questionable power for detecting an effect of job insecurity on suicide. Furthermore, our power calculations indicated that limiting the follow-up time even further would have resulted into an overly high risk of making a Type II error. Information about job insecurity was self-reported, thus not influenced by recall bias and also more reliable than when using psychological autopsy methods (ie, relying on information collected retrospectively from family members, relatives or friends of the deceased). Furthermore, we investigated a large sample and used information on exposure and outcome from different data sources, minimizing the risk of common method variance which may create spurious associations and limit causal interpretations.

As only those who seek care are registered in the national health registers, an under-ascertainment in assessment of suicide attempts is, however, likely. It is further difficult to accurately identify the correct intent of injury or death. This may contribute to misclassification of the outcome, which if non-differential most often drives the estimates towards the null. Including death with undetermined intent is advised as it reduces both geographical variations as well as time trends in ascertainment and classification of suicide (46), but risks increasing the number of suicides which are false positive. The findings were not strengthened when limiting the analysis to suicides ascertained by a diagnosis of self-inflicted harm, but the latter analyses were limited by reduced statistical power. The assessment of job insecurity was based on one single dichotomous item, potentially less precise than multi-item measures. The single-item measure on job insecurity could also have underestimated the association between job insecurity and suicide as effect sizes for single-item measures of job insecurity tend to be smaller compared to estimates from scales with multiple items (47). In addition, as the data source used (SWES) only had one measurement of insecurity per participant, it was not possible to consider insecurity as a time-varying exposure. Moreover, the MSC model is considered to deal efficiently with confounding and can handle covariates both acting as confounders and mediators, thus making causal interpretations possible. Unfortunately, though, we were only able to include covariates from baseline and may not have fully benefited from the MSC advantage of handling time-varying confounders. This may have limited the possibility to get valid estimates when additionally considering factors that may be both confounders and mediators of the relationship, such as mental disorders which may be important to consider when studying suicidal behaviors. Hence, the sensitivity analyses considering possible mediators could underestimate the association of interest. However, in absence of exposure–confounder feedback effects, estimates from MSC are similar to traditional Cox model estimates (48). Analyses based on the same data using traditional Cox regression gave similar estimates, but this method is slightly less conservative than the MSC method. Although many sociodemographic confounders were included in the analysis and certain work characteristics that are seldom considered, there may still be residual confounding. For instance, it cannot be ruled out that the observed elevated risk for suicide death by perceived job insecurity crossed over unity due to unmeasured confounding. The estimate suggests that the excess risk is small and the lower confidence bound is close to one. For example, time invariant characteristics such as genetic predisposition, personality, family history and childhood experiences, which may be associated with both perceptions of job insecurity and suicidal behavior, were not possible to include in the analyses. Personality traits in common for both job insecurity and suicide such as neuroticism may for example have biased the findings, although neuroticism may also be a consequence of job insecurity rather than an antecedent (49, 50). Moreover, prior studies have found job insecurity to vary by socioeconomic position. Low skill occupations and a low educational level are associated with greater job insecurity while higher socioeconomic positions according to occupation and educational level are associated with lower job insecurity (51). A similar pattern has been observed with regard to suicide risk (52). In the present study, we were able to account for education and income, while we had no information about an individual’s occupation. Hence, we cannot preclude that the study findings are partly attributable to characteristics associated with a certain occupation or with selection effects. Finally, job insecurity is likely to be particularly common in precarious and temporary employment. These employment groups as well as part-time workers and self-employed are likely to be less represented among the participants in our study, limiting the generalizability of the findings. This may have contributed to underestimation of associations.

### Concluding remarks

In this study, job insecurity was associated with an elevated risk of death by suicide but not suicide attempts. The study provides empirical support from individual level data that job insecurity may be a relevant stressor associated with suicide. This suggests that reducing insecurity may reduce suicide risk. Policies and practices that either limit job insecurity or counterbalance the negative consequences of job insecurity, for example monetary security or increased employability, could reduce suicide deaths. Mitigating or compensating for job insecurity may be particularly important during times like these of the COVID-19 pandemic when insecurity increases.

### Funding

This work was supported by the Swedish Council for Health, Working Life, and Welfare [#2009-1758] and the Swedish Research Council [#2017-00624, # 2013-1645, # 2013-1645].

### Conflict of interest

No conflicts of interest to declare.

### Ethical standards

The study has received ethical approval by the Regional Research Ethics Board in Stockholm, document numbers: 2012/373-31/5, 2013/2173–32, 2015/2187–32, 2015/2298–32, and 2017/2535-32. Participants of the Swedish Work Environment Survey received written information on the survey, by answering and returning the survey respondents confirmed their informed consent.

## Supplementary material

Supplementary material
