# Supplementary material for: Perceived job insecurity and risk of suicide and suicide attempts: a study of men and women in the Swedish working population
Source: Scand J Work Environ Health. 2022 Apr 29;48(4):293–301. doi: 10.5271/sjweh.4015 (PMC9524162; doi:10.5271/sjweh.4015)
Supplement: Supplementary material [file SJWEH-48-293-S001.pdf]

Perceived job insecurity and risk of suicide and suicide attempts: a study of men and women in the Swedish working population[1]

by Sandra Blomqvist, MSc,[2] Marianna Virtanen, PhD, Anthony D LaMontagne, ScD, Linda L Magnusson Hanson, PhD

1. Supplementary tables
2. Correspondence to: Sandra Blomqvist, Stress Research Institute, Stockholm University, SE-106 91 Stockholm, Sweden. [E-mail: sandra.blomqvist@su.se]

Table S1. Distribution of sociodemographic factors according to job insecurity, by sex.

		Men						Women					
		Not exposed			Exposed			Not exposed			Exposed		
		N	%	mean (SD)	N	%	/mean (SD	N	%	mean (SD)	N	%	/mean (SD
**Age**				41 (12)			40 (12)			42 (12)			40 (11)
**Birth country**	Nordic countries	25920	96		3936	96		29050	97		4330	96	
	Elsewhere	942	4		169	4		1030	3		194	4	
**Family situation**	Married/living with partner w. children	13312	50		2107	49		13875	46		2147	48	
	Married/living with partner w/o children	4303	16		557	14		5848	19		645	14	
	Single/divorced/separated/widowed w. children	1166	4		176	4		3095	10		538	12	
	Single/divorced/separated/widowed w/o children	8081	30		1355	33		7262	24		1194	26	
**Education**	Compulsory school (≤9 yr)	6064	23		888	22		5447	18		804	18	
	Gymnasium (10-12 yr)	13091	49		2287	56		14799	49		2550	56	
	University (≥13 yr)	7707	29		930	23		9834	33		1170	26	
**Yearly income from work in SEK**				238103 (124340)			209258 (120595)			165555 (85832)			144048 (76657)
**Job demands**				1.6 (1.3)			1.5 (1.2)			1.8 (1.3)			1.7 (1.3)
**Job control**				2.7 (1.2)			2.4 (1.3)			2.4 (1.3)			2.1 (1.3)
**Support at work**				1.7 (1.0)			1.8 (1.1)			1.5 (0.8)			1.6 (0.9)

Table S2. Results from traditional Cox regression analyses estimating the relationship between job insecurity and suicide attempts and suicide mortality.

			Basic model[b]		Adjusted model[c]	
	N	cases	**HR**	**CI**	**HR**	**CI**
**Suicide[a]**						
All	65571	170	**1.56**	**1.06-2.28**	**1.51**	**1.03-2.20**
Men	30967	114	1.38	0.85-2.23	1.33	0.82-2.15
Women	34604	56	**1.91**	**1.03-3.56**	**1.88**	**1.01-3.50**
**Suicide attempts[a]**						
All	65330	896	1.05	0.87-1.27	1.03	0.85-1.24
Men	30857	439	1.20	0.93-1.55	1.18	0.92-1.52
Women	34473	457	0.91	0.69-1.19	0.88	0.67-1.16

a) Results presented in Hazard Ratio (HR) and 95% Confidence Intervals (CI), made bold when significant at an alpha level of 0.05

b) Unadjusted model but with age as time scale

c) Adjusted for sex, birth country, family situation, and educational level

Table S3. Association between job insecurity and suicide or suicide attempts with 10 years of follow-up.[a]

	N	cases	**HR**	**CI**
**Suicide[b]**				
All	65571	84	**1.93**	**1.14-3.27**
Men	30967	49	1.79	0.85-3.78
Women	34604	35	2.10	0.88-5.02
**Suicide attempts[b]**				
All	65557	476	0.99	0.76-1.29
Men	30960	234	1.26	0.90-1.77
Women	34597	242	0.75	0.48-1.16

a) Estimates obtained from Marginal Structural Cox regression analyses with inverse probability weighting considering sex, birth country, family situation, and educational level

b) Results presented in Hazard Ratio (HR) and 95% Confidence Intervals (CI) obtained from bootstrapping, in relation to unexposed, made bold when significant at an alpha level of 0.05

Table S4. Results from traditional Cox regression analyses estimating the relationship between job insecurity and suicide attempts and suicide mortality with a 10 year follow-up time.[a]

			Basic model[b]		Adjusted model[c]	
	N	cases	**HR**	**CI**	**HR**	**CI**
**Suicide[a]**						
All	65571	84	**2.01**	**1.20-3.35**	**1.96**	**1.17-3.27**
Men	30967	49	1.95	1.00-3.82	1.88	0.96-3.68
Women	34604	35	2.05	0.93-4.53	2.06	0.93-4.57
**Suicide attempts[a]**						
All	65557	476	0.99	0.76-1.30	0.96	0.74-1.26
Men	30960	234	1.27	0.89-1.79	1.24	0.88-1.76
Women	34597	242	0.74	0.49-1.13	0.72	0.48-1.09

a) Results presented in Hazard Ratio (HR) and 95% Confidence Intervals (CI), made bold when significant at an alpha level of 0.05

b) Unadjusted model but with age as time scale

c) Adjusted for sex, birth country, family situation, and educational level

Table S5. Association between job insecurity and suicide or suicide attempts ascertained by a diagnosis of self-inflicted harm (excluding those of undetermined intent).[a]

	N	cases	**HR**	**CI**
**Suicide[b]**				
All	65571	147	1.35	0.88-2.07
Men	30967	99	1.23	0.70-2.16
Women	34604	48	1.56	0.71-3.43
**Suicide attempts[b]**				
All	65329	405	0.90	0.66-1.22
Men	30856	174	1.09	0.71-1.68
Women	34473	231	0.77	0.51-1.15

a) Estimates obtained from Marginal Structural Cox regression analyses with inverse probability weighting considering sex, birth country, family situation, and educational level

b) Results presented in Hazard Ratio (HR) and 95% Confidence Intervals (CI) obtained from bootstrapping, in relation to unexposed.
